# Acetylation Rather than H50Q Mutation Impacts the Kinetics of Cu(II) Binding to α‐Synuclein

**DOI:** 10.1002/cphc.202100651

**Published:** 2021-10-14

**Authors:** Xiangyu Teng, Alena Sheveleva, Floriana Tuna, Keith R. Willison, Liming Ying

**Affiliations:** ^1^ National Heart and Lung Institute Imperial College London White City Campus London W12 0BZ UK; ^2^ Department of Chemistry Imperial College London White City Campus London W12 0BZ UK; ^3^ Department of Chemistry The University of Manchester Oxford Road Manchester M13 9PL UK

**Keywords:** α-synuclein, copper, fluorescence spectroscopy, kinetics, coordination modes

## Abstract

The interaction between α‐synuclein (αSyn) and Cu^2+^ has been suggested to be closely linked to brain copper homeostasis. Disruption of copper levels could induce misfolding and aggregation of αSyn, and thus contribute to the progression of Parkinson's disease (PD). Understanding the molecular mechanism of αSyn‐Cu^2+^ interaction is important and controversies in Cu^2+^ coordination geometry with αSyn still exists. Herein, we find that the pathological H50Q mutation has no impact on the kinetics of Cu^2+^ binding to the high‐affinity site of wild type αSyn (WT‐αSyn), indicating the non‐involvement of His50 in high‐affinity Cu^2+^ binding to WT‐αSyn. In contrast, the physiological N‐terminally acetylated αSyn (NAc‐αSyn) displays several orders of magnitude weaker Cu^2+^ binding affinity than WT‐αSyn. Cu^2+^ coordination mode to NAc‐αSyn has also been proposed based on EPR spectrum. In addition, we find that Cu^2+^ coordinated WT‐αSyn is reduction‐active in the presence of GSH, but essentially inactive towards ascorbate. Our work provides new insights into αSyn‐Cu^2+^ interaction, which may help understand the multifaceted normal functions of αSyn as well as pathological consequences of αSyn aggregation.

## Graphical abstract

**Understanding the molecular mechanism of αSyn‐Cu^2+^ interaction**: From a kinetic perspective, His50 of αSyn is irrelevant to high‐affinity Cu^2+^ binding, while N‐terminal acetylation attenuates Cu^2+^ binding affinity of αSyn around four orders of magnitude. Reduction kinetics of Cu^2+^ coordinated αSyn is also reported.

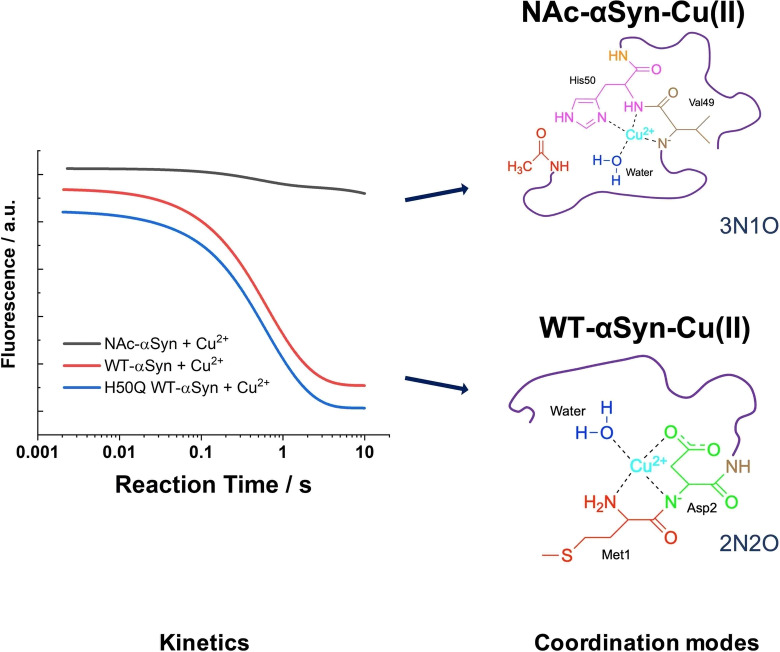

## 1 Introduction

Parkinson's disease (PD) is the second most common neurodegenerative disease, currently affecting more than 10 million people worldwide.^[1]^ Two characteristic features of PD are the loss of dopaminergic neurons in the *substantia nigra* and intracellular deposition of Lewy bodies (LBs) predominantly composed of α‐synuclein (αSyn). αSyn is a ∼14 kDa intrinsically disordered protein (IDP) mainly located in presynaptic terminals with an abundance equivalent to ∼50 μM free concentration.^[2]^ Abnormal aggregation of αSyn is believed to associate with the pathology of PD.^[3]^ However, the precise mechanism that induces the abnormal aggregation is yet to be fully established. Whereas increasing evidence has indicated that αSyn mutation,[^4^, ^5^] posttranslational modification^[6]^ and metal ion interactions[^7^, ^8^, ^9^] can all accelerate αSyn aggregation.

Single‐point mutations within αSyn, including A30P, E46K, H50Q, G51D and A53T/E/V, have been discovered to be responsible for the onset of familial PD (FPD).^[10]^ These mutations display various effects on the fibrillisation rate of αSyn.^[11]^ Notably, the H50Q mutation was able to significantly reduce the solubility of αSyn and promote αSyn fibrillisation.^[12]^

**Figure 1:**
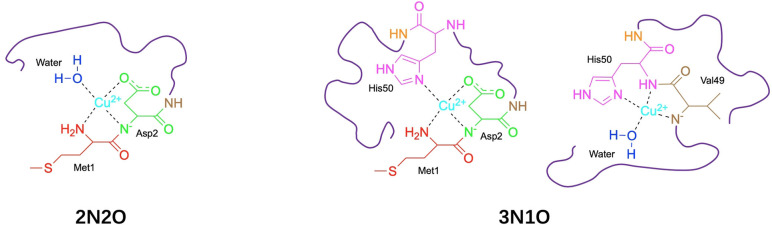
Proposed coordination modes of αSyn‐Cu(II) complex at physiological pH. Coordinations involving N‐terminal methionine (Met1) and aspartic acid (Asp2) display high‐affinity Cu^2+^ binding, including the 2N2O mode and one of the 3N1O modes (left). Another 3N1O mode (right) displays low‐affinity Cu^2+^ binding. Adapted from Ref. [20].

Over the past two decades, a series of transition metal ions has been proven to be able to accelerate the misfolding of αSyn.^[13]^ Given that the physiological concentrations of these ions are typically in the nanomolar to low micromolar regime, only Cu^2+^ would be able to exhibit a pronounced acceleration effect due to its higher affinity with αSyn in comparison to other metal ions.[^14^, ^15^] Moreover, since Cu^2+^ is redox active, enriched Cu^2+^ in αSyn aggregates can locally promote the production of reactive oxygen species (ROS) which damage neurons.[^16^, ^17^] To date, three regions of αSyn have been suggested to interact with Cu^2+^, which are located at N‐terminus (Met1 and Asp2), His50 and Asp121, respectively.^[18]^ Five possible Cu^2+^ coordination modes have been proposed in the pH range between 5.0 and 7.4.[^19^, ^20^] Among them, three coordination modes at physiological pH are of considerable interest (Figure 1), but the existence of 3N1O modes centred at His50 is currently under dispute. De Ricco *et al*. suggested that the three modes can switch between each other depending on Cu^2+^ concentration and pH.^[20]^ In addition, studies on the peptides obtained from the αSyn sequence confirmed that His50 can be involved in the coordination of Cu^2+^.[^21^, ^22^] However, the work by Tian and co‐workers ruled out the presence of 3N1O modes in αSyn‐Cu^2+^ coordination under physiological conditions,^[23]^ and a biophysical study of Cu^2+^ binding to the αSyn fragments found no evidence of the participation of His50 in strong Cu^2+^ binding.^[24]^ Since His50 also plays an important role in FPD pathogenesis, elucidating this issue will not only enhance the fundamental understanding of the interaction between Cu^2+^ and αSyn, but also shed new insights into the pathology of FPD induced by the H50Q variant.

Human αSyn in its physiological state is predominantly N‐terminal acetylated (NAc),[^25^, ^26^] but the significance of NAc for αSyn has not yet been fully established. Apart from moderately enhanced lipid membrane binding for NAc‐αSyn in comparison to wild type (WT) αSyn, one notable observation is that N‐terminal acetylation abolishes the high‐affinity Cu^2+^ binding site (M1D2), thus weakening the binding between Cu^2+^ and αSyn.^[27]^ Nevertheless, Cu^2+^ affinity has not been reported for NAc‐αSyn.

Kinetic techniques have been employed recently in several studies to give insights into IDP‐Cu^2+^ interactions.[^28^, ^29^, ^30^, ^31^] In brain, the existence of labile Cu^2+^ released in synaptic cleft during neuronal excitation is transient. Therefore, kinetic techniques are more powerful and better suited to investigate αSyn‐Cu^2+^ interaction compared to equilibrium or steady‐state measurements. Here we report the effects of the H50Q mutation and N‐terminal acetylation on Cu^2+^ binding to αSyn from kinetic perspective. Highly sensitive fluorescent probe was used to detect fast reaction kinetics as reported previously.[^29^, ^30^, ^31^] We have found that His50 in αSyn is not involved in high‐affinity Cu^2+^ binding, whereas N‐terminal acetylation reduces the Cu^2+^ binding affinity of αSyn by approximately four orders of magnitude. Furthermore, we have shown by kinetic measurements that αSyn‐Cu(II) complex can be readily reduced under physiological conditions by glutathione (GSH) instead of ascorbate.

## 2 Results and Discussion

**Figure 2:**
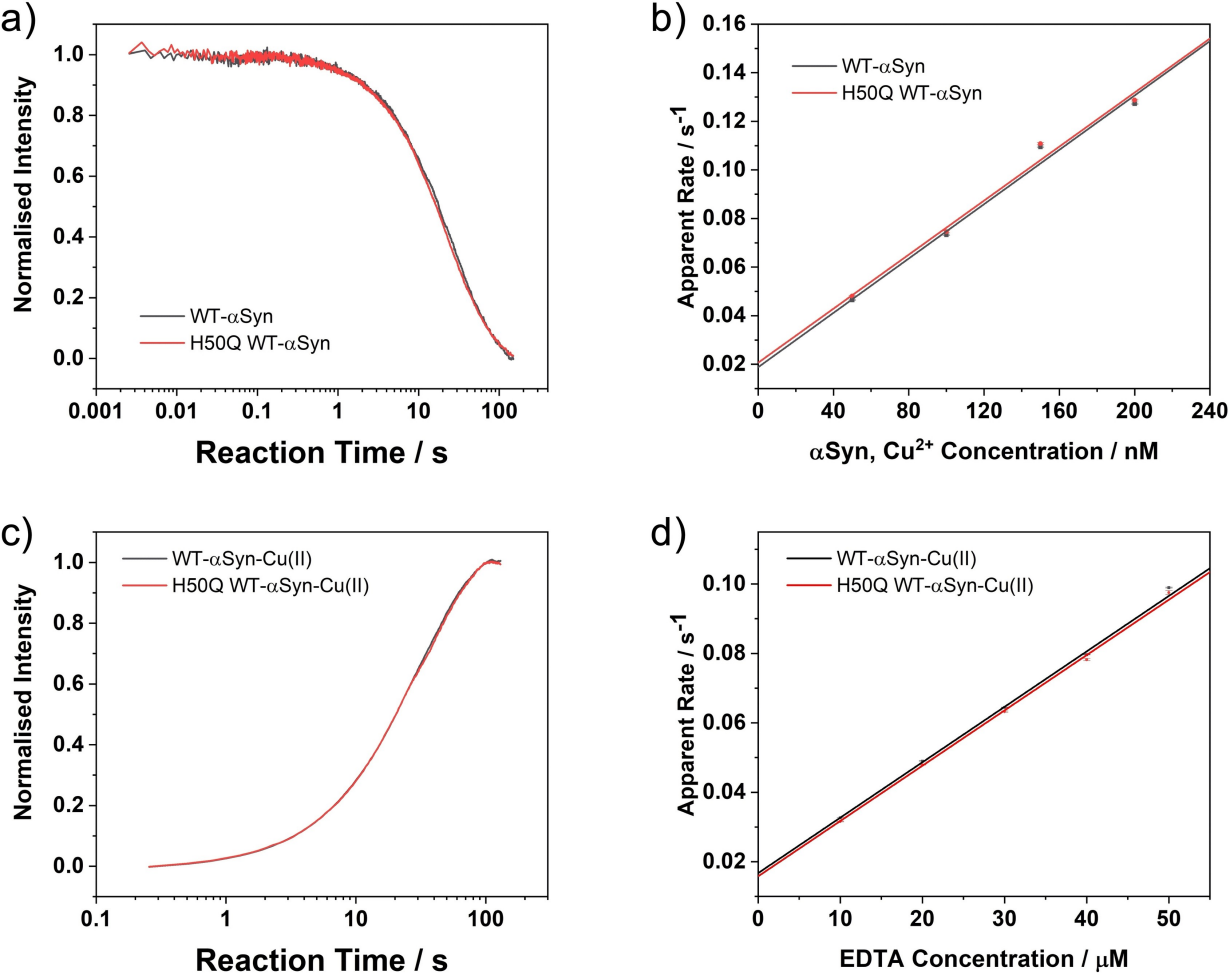
Comparison of Cu^2+^ binding kinetics between WT‐αSyn and H50Q WT‐αSyn, as well as reaction kinetics of the two corresponding Cu^2+^ coordinated αSyn complexes with EDTA. a) Reaction traces of 50 nM Cu^2+^ binding to 50 nM WT‐αSyn and H50Q WT‐αSyn. b) Apparent Cu^2+^ association rates with WT‐αSyn and H50Q WT‐αSyn. c) Reaction traces of 50 nM WT‐αSyn‐Cu(II) and H50Q WT‐αSyn‐Cu(II) with 10 μM EDTA. d) Apparent reaction rates of WT‐αSyn‐Cu(II) and H50Q WT‐αSyn‐Cu(II) with EDTA. All measurements were performed in 50 mM HEPES buffer with 100 mM NaCl at 298 K (pH 7.5).

To investigate whether the H50Q mutation of αSyn can affect Cu^2+^ binding to αSyn, stopped flow kinetic measurements were performed. Cu^2+^ binding to Alexa 488 labelled WT‐αSyn and H50Q WT‐αSyn were carried out first under 1 : 1 mixing ratio of labelled protein to Cu^2+^. The Cu^2+^ association rate constants (*k*_on_) to both constructs were then derived. Representative raw traces are shown in Figure 2a and Figure S1. The apparent Cu^2+^ association rates (*k*_on(App)_) were determined by fitting the reaction traces (fitting functions are described in Methods), and then plotting these rates against Cu^2+^ concentration as shown in Figure 2b. *k*_on_ of Cu^2+^ binding to WT‐αSyn and H50Q WT‐αSyn in HEPES buffer and 100 mM NaCl were determined from the slopes of the linear fits, which are 5.6(5)×10^5^ M^−1^ s^−1^ and 5.5(3)×10^5^ M^−1^ s^−1^, respectively. Such close values of *k*_on_ and virtually identical reaction traces strongly suggest that Cu^2+^ binding to both WT‐αSyn and H50Q WT‐αSyn share the same mechanism at low αSyn concentration, i. e., His50 of WT‐αSyn is not involved in Cu^2+^ binding under such conditions.

Next, the reactions of Alexa 488 labelled WT‐αSyn‐Cu(II) and H50Q WT‐αSyn‐Cu(II) complexes with EDTA were performed. If His50 was involved in Cu^2+^ coordination as proposed,^[20]^ two pH‐dependent coordination species differing in reactivity with EDTA should be observed in a similar manner to that reported for Aβ.^[31]^ 50 nM labelled WT‐αSyn and labelled H50Q WT‐αSyn samples were both pre‐mixed with 50 nM CuCl_2_ to form the complexes which were then reacted with EDTA in various concentrations. As shown in Figure 2c and Figure S2, the raw traces for the two complexes are almost identical. As expected, very similar reaction rate constants for Cu^2+^ extraction from WT‐αSyn‐Cu(II) and H50Q WT‐αSyn‐Cu(II), 0.017(4) s^−1^ and 0.015(5) s^−1^ respectively, were observed (Figure 2d). This kinetic evidence suggests that the Cu^2+^ coordination modes of WT‐αSyn and H50Q WT‐αSyn are virtually identical. In addition, reactions of WT‐αSyn‐Cu(II) with EDTA under different pH (5.5, 6.5 and 7.5) showed no evidence of the presence of more than one species (Figure S3). Therefore, the involvement of His50 in 3N1O coordination modes for WT‐αSyn is questionable.

**Figure 3:**
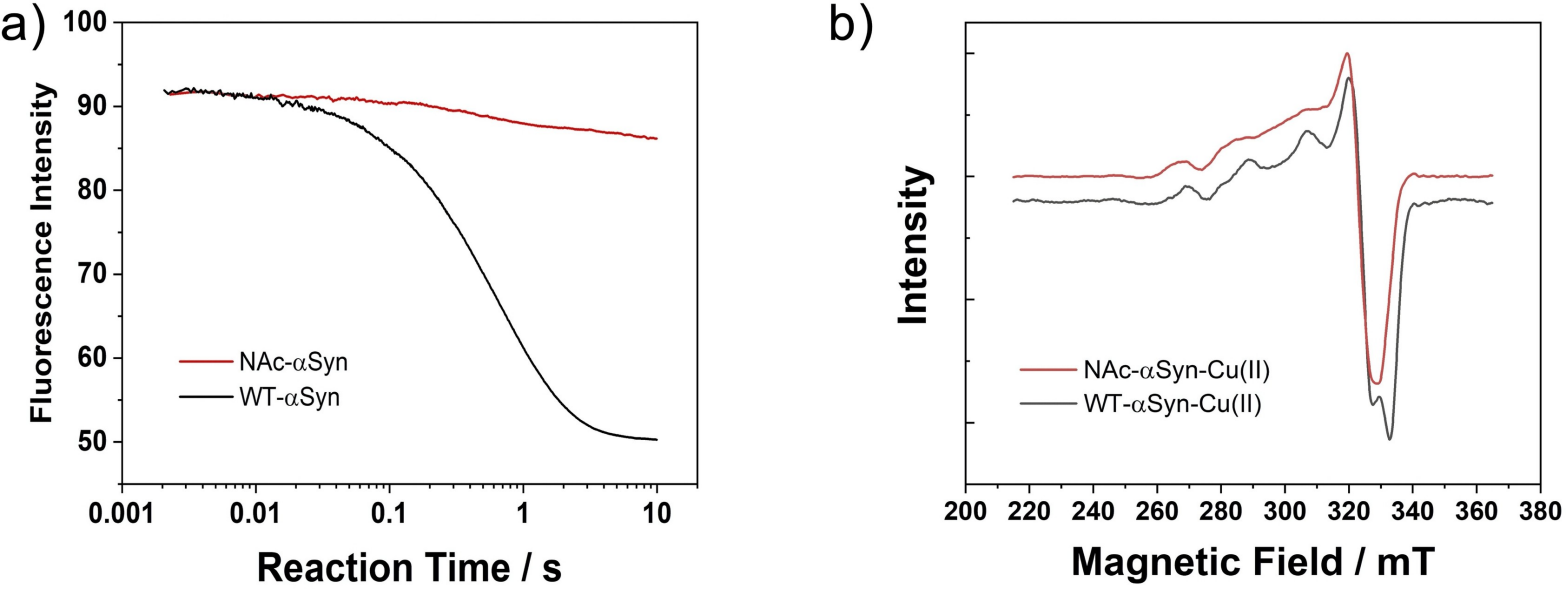
Differences between Cu^2+^ binding to WT‐αSyn and NAc‐αSyn. a) Kinetic traces of Cu^2+^ binding to NAc‐αSyn and WT‐αSyn (25 nM αSyn, 500 nM Cu^2+^). The measurements were performed in 50 mM HEPES buffer with 100 mM NaCl at 298 K (pH 7.5). b) X‐band EPR spectra of Cu^2+^ bound on NAc‐αSyn and WT‐αSyn (50 μM αSyn, 50 μM Cu^2+^). The measurements were performed in 50 mM HEPES buffer with 100 mM NaCl and 25 % glycerol (pH 7.5). The spectra were recorded at 20 K, 9.4 GHz.

**Figure 4:**
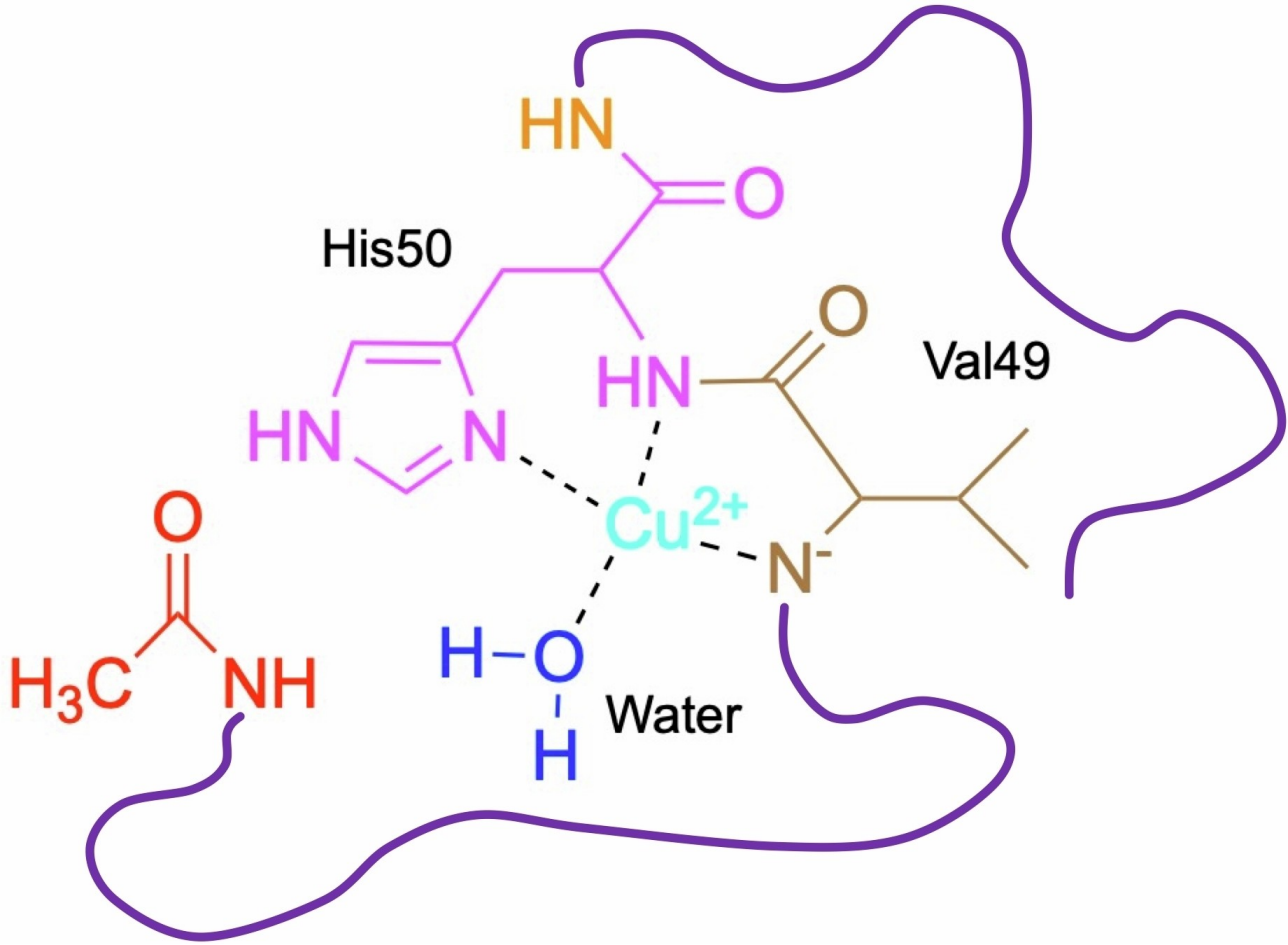
Proposed Cu^2+^ coordination mode for NAc‐αSyn.

So far, it turns out that H50Q mutation has no observable effect on the high‐affinity binding between Cu^2+^ and αSyn. However, N‐terminal acetylation can significantly impact the interactions as it would destroy the high‐affinity Cu^2+^ binding site at the N‐terminus of αSyn. Such an impact can be detected by either monitoring the kinetics of Cu^2+^ binding to the protein or X‐band EPR measurements of Cu^2+^ coordination mode of the protein‐Cu(II) complex, as shown in Figure 3. Once the N‐terminal Cu^2+^ binding site (M1D2) is abolished, two remaining low‐affinity binding sites centred at His50 and Asp121 respectively, would be in charge.[^32^, ^33^] According to the Peisach‐Blumberg plot^[34]^ (Figure S4), the *g*_∥_ factor and hyperfine coupling constant (*A*_∥_) of NAc‐αSyn‐Cu(II), derived by spectral simulation (shown in Table S1), were found to be in good agreement with a 3N1O mode. His50 could be the central residue of this mode, as an Asp121 centred form was proposed to adopt the 4O mode and exist in acidic environment.^[19]^ The proposed Cu^2+^ coordination mode of NAc‐αSyn is illustrated in Figure 4.

**Figure 5:**
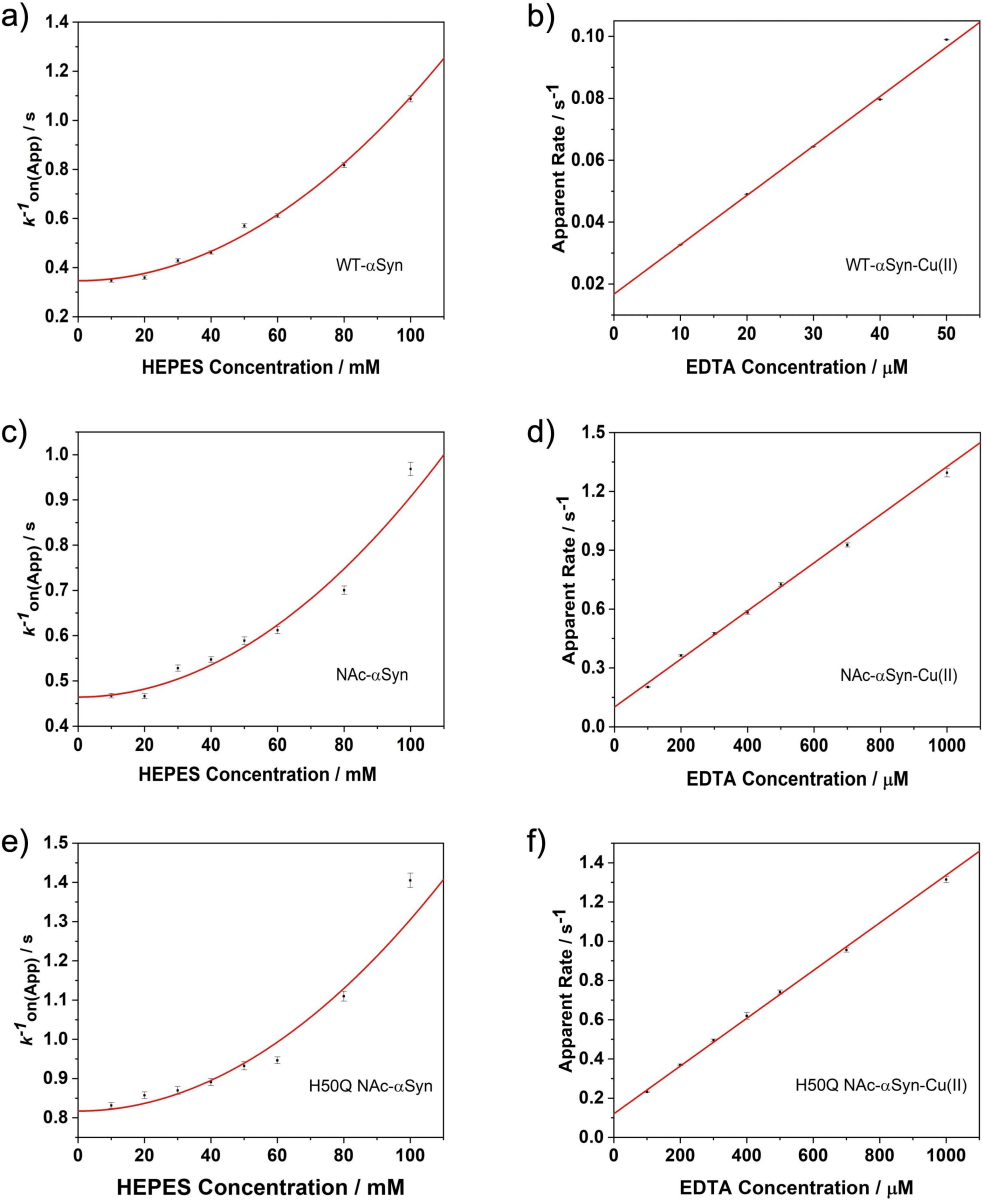
Kinetics of Cu^2+^ binding to αSyn and Cu^2+^ extraction from αSyn‐Cu(II) complex by EDTA. a, c, e) are HEPES dependence of *k*_on(App)_ for Cu^2+^ binding to WT‐αSyn ([Cu^2+^]=500 nM), NAc‐αSyn ([Cu^2+^]=500 μM) and H50Q NAc‐αSyn ([Cu^2+^]=500 μM), respectively. b, d, f) are apparent reaction rates of WT‐αSyn‐Cu(II), NAc‐αSyn‐Cu(II) and H50Q NAc‐αSyn‐Cu(II) with EDTA, respectively.

The kinetic parameters for the interactions of Cu^2+^ with WT‐αSyn and NAc‐αSyn were determined to further evaluate the effect of N‐terminal acetylation on Cu^2+^ binding. The apparent association rate constants of Cu^2+^ binding to αSyn were first determined. 25 nM labelled WT‐αSyn was reacted with 500 nM Cu^2+^ under various HEPES concentrations to obtain the HEPES‐independent binding rate constant *k*_on_. HEPES is good biological buffer but still a weak Cu^2+^ chelator, therefore a correction must be made to account for the effective concentration of free Cu^2+^.^[35]^ Due to the fast preequilibrium (∼μs timescale) between the binding of free Cu^2+^ with HEPES and the dissociation of the resulting complex, the correction factor for binding kinetics (∼ms–s timescale) is expected to be rather small in comparison to that for the binding equilibrium constant. Here we derived buffer‐independent *k*_on_ from the intercept of the fitting, which used an empirically chosen zero‐centred parabola.^[31]^ In case of NAc‐αSyn, since the reactions are relatively slow, 25 nM labelled protein was reacted with 500 μM Cu^2+^ to accelerate the binding. The raw traces are shown in Figure S5, while the results are shown in Figure 5a and 5c. The buffer independent *k*_on_ values are 5.7(1)×10^6^ M^−1^ s^−1^ and 4.3(2)×10^3^ M^−1^ s^−1^ for WT‐αSyn and NAc‐αSyn, respectively. Acetylation reduces the Cu^2+^ binding rate constant by approximately three orders of magnitude.

The spontaneous Cu^2+^ dissociation rate constants (*k*_off_) from the complexes were subsequently determined. The labelled WT‐αSyn and Cu^2+^ were pre‐mixed at 50 nM, while labelled NAc‐αSyn was pre‐mixed with unlabelled NAc‐αSyn stock solution to prepare a 10 μM protein solution containing 50 nM labelled protein and then mixed with 10 μM Cu^2+^. The mixtures were subsequently reacted with various concentrations of EDTA. The raw traces are shown in Figure S6. The dissociation rate constants were determined from the intercepts of linearly fitted apparent rates (Figure 5b and 5d), which are 0.017(4) s^−1^ and 0.10(1) s^−1^ for WT‐αSyn‐Cu(II) and NAc‐αSyn‐Cu(II), respectively. *k*_off_ of Cu^2+^ dissociation from NAc‐αSyn‐Cu(II) is approximately six times faster than that of WT‐αSyn‐Cu(II). These *k*_off_ values together with *k*_on_ determined above, gave the equilibrium dissociation constants (*K*_d_) of 3.0(7) nM and 23(3) μM for Cu^2+^ binding to WT‐αSyn and NAc‐αSyn, respectively. Therefore, N‐terminal acetylation weakens the Cu^2+^ binding affinity of αSyn around four orders of magnitude. In addition, the second‐order rate constants for the reaction of αSyn‐Cu(II) complexes with EDTA were determined from the slopes of Figure 5b and 5d, which are 1.60(2)×10^3^ M^−1^ s^−1^ and 1.23(5)×10^3^ M^−1^ s^−1^ for WT‐αSyn‐Cu(II) and NAc‐αSyn‐Cu(II), respectively.

Since His50 could be the central Cu^2+^ binding site of NAc‐αSyn, analogue kinetic experiments were conducted to understand how His50 removal affects Cu^2+^ binding to NAc‐αSyn. In these experiments, the pathological mutation H50Q was chosen and the binding kinetics of the labelled H50Q NAc‐αSyn with Cu^2+^ was investigated under the conditions identical to the studies with NAc‐αSyn as described above. *k*_on_ (Figure 5e) and *k*_off_ (Figure 5f) are determined to be 2.4(1)×10^3^ M^−1^ s^−1^ and 0.12(1) s^−1^, respectively, giving the *K*_d_ of 50(6) μM for Cu^2+^ binding to H50Q NAc‐αSyn, a reduction of two‐fold in comparison to NAc‐αSyn. Therefore, His50 is the preferred Cu^2+^ binding site after acetylation, in agreement with the literature work.^[27]^ The second‐order rate constant for the reaction with EDTA was also determined, which is 1.22(2)×10^3^ M^−1^ s^−1^, similar to that of NAc‐αSyn‐Cu(II). Kinetic parameters (association and dissociation rate constant) and thermodynamic parameter (equilibrium dissociation constant) of the interactions between αSyn and Cu^2+^ are listed in Table 1.

**Table 1:** Kinetic and thermodynamic parameters for the interactions between αSyn and Cu^2+^.

	WT‐αSyn	NAc‐αSyn	H50Q NAc‐αSyn
*k*_on_/M^−1^ s^−1^	5.7(1)×10^6^	4.3(2)×10^3^	2.4(1)×10^3^
*k*_off_/s^−1^	0.017(4)	0.10(1)	0.12(1)
*K*_d_/μM	3.0(7)×10^−3^	23(3)	50(6)

**Figure 6:**
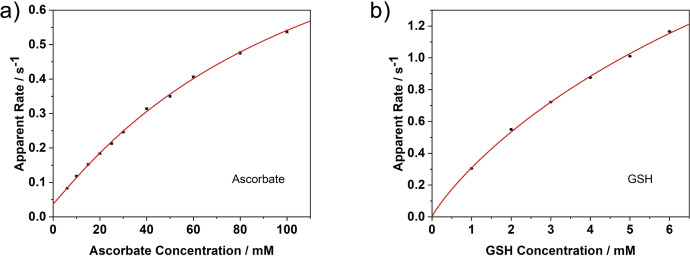
Reduction kinetics of WT‐αSyn‐Cu(II) complex under various concentrations of a) ascorbate and b) GSH.

Finally the reduction kinetics of WT‐αSyn‐Cu(II) was studied. Two common cellular antioxidants, ascorbate and glutathione (GSH), were selected as reductants. As Cu^2+^ is a paramagnetic ion with a single unpaired electron, it can readily interact with a fluorophore's first excited state which also possesses a single unpaired electron, hence quenching the fluorescence of the fluorophore. However, once Cu^2+^ is reduced to Cu^+^, it no longer possesses an unpaired electron, thus the fluorescence of the dye label will recover. Our measurement was designed based on this principle. 50 nM labelled WT‐αSyn was pre‐mixed with 50 nM Cu^2+^ to form WT‐αSyn‐Cu(II) complex, which was then reacted with sodium ascorbate or GSH at various concentrations. The apparent reduction rates as a function of antioxidant concentration, derived from the raw traces in Figure S7, are shown in Figure 6. Strikingly, the reduction reaction in the presence of ascorbate is quite slow even at 10–100 mM ascorbate concentration. In contrast, the reduction in the presence of GSH is much faster. Since under physiological conditions, GSH has much higher concentration than ascorbate, the contribution to the reduction of the complex from ascorbate is expected to be negligible.

To confirm whether His50 impacts Cu^2+^ binding with WT‐αSyn, we not only compared the Cu^2+^ binding and dissociation rate constants of WT‐αSyn and H50Q WT‐αSyn, but also investigated the pH dependence of the binding reaction involving Cu^2+^ and WT‐αSyn. Ultrasensitive stopped flow kinetic experiments, conducted via monitoring the variation in fluorescence from a bright fluorophore induced either by Cu^2+^ binding or dissociation, enabled us to identify similar Cu^2+^ binding rate constants between the two αSyn constructs and observe pH‐independent reaction profile of WT‐αSyn. Both results indicate that His50 is essentially irrelevant for Cu^2+^ binding to WT‐αSyn when only the high‐affinity binding site is involved in the binding reaction under the reaction conditions investigated. Consequently, the pathological H50Q mutation has no impact on the kinetics of Cu^2+^ binding to the high‐affinity site of αSyn. Such observation is in good agreement with the result obtained by Tian *et al*.,^[23]^ but contradictory to that of De Ricco and co‐workers.^[20]^ The reason could be that the experimental concentration of αSyn in the latter study was so high that a Cu^2+^ bridged ternary complex was generated.^[23]^

In contrast, using an analogous method, the Cu^2+^ binding affinity of NAc‐αSyn (*K*_d_=23(3) μM) was determined to be around four orders of magnitude weaker than that of WT‐αSyn (*K*_d_=3.0(7) nM). So far, *K*_d_ values reported for Cu^2+^ binding to WT‐αSyn as determined by different techniques vary between 0.1 nM and 0.7 μM.^[36]^ The *K*_d_ for Cu^2+^ binding to WT‐αSyn determined here lies well within this range. However, there is no reported value for the *K*_d_ of Cu^2+^ binding to NAc‐αSyn for comparison. Independent and carefully designed tyrosine fluorescence titration experiments^[37]^ are desirable to confirm the affinity values determined in this work. We also found that His50 of NAc‐αSyn now dominates this weak binding thanks to the abolishment of N‐terminal high‐affinity binding site by acetylation. Finally, we found that WT‐αSyn‐Cu(II) complex is hard to be reduced by ascorbate, but can be effectively reduced by GSH.

Cu^2+^ concentrations in synaptic cleft can transiently reach up to 15 μM,^[38]^ while intracellular αSyn concentration is reported around 50 μM.^[2]^ According to the binding rate constant determined by this work, the association between Cu^2+^ and WT‐αSyn is expected to occur on the millisecond timescale, which would significantly dampen the magnitude of the spike of the released Cu^2+^ concentration. As NAc‐αSyn is the predominant αSyn form, orders of magnitude weaker binding between Cu^2+^ and the physiological NAc‐αSyn observed here may implicate the importance of N‐terminal acetylation of WT‐αSyn: which is to prevent the depletion of labile Cu^2+^. Labile Cu^2+^ is involved in the regulation of neurotransmission,^[39]^ acetylation would thus safeguard the neurotransmission, and also mitigate the risk of Cu^2+^ induced αSyn aggregation. Moreover, it has been reported that NAc‐αSyn readily binds to Cu^+^ (*K*_d_=12(4) μM).^[40]^ Therefore, physiological NAc‐αSyn could act as a relay in the copper transport chain which might absorb transiently excess Cu^+^ out of the reducing environment and ship to copper transporters. Consequently, dysfunction of N‐terminal acetylation of αSyn would disrupt the two potential functional roles of αSyn. However, H50Q mutation seems not to impact Cu^2+^ binding kinetics of WT‐αSyn, strongly suggesting that His50 is not involved in high‐affinity Cu^2+^ binding. There is evidence that Cu^2+^ can promote the aggregation of H50Q WT‐αSyn even more significantly than that of WT‐αSyn,^[41]^ suggesting that His50 in WT‐αSyn would not play a predominant role in Cu^2+^ binding. Whereas for NAc‐αSyn, the absence of His50 reduces Cu^2+^ affinity by two folds, which is in good agreement with the previous study.^[32]^ In addition, WT‐αSyn‐Cu(II) complex is not as redox‐active as WT‐Aβ‐Cu(II) complex which can be reduced by 0.1 mM ascorbate in 10 s.^[42]^ Therefore, ROS may not be easily generated via αSyn‐Cu(II) even though a small amount of WT‐αSyn‐Cu(II) might be present physiologically.

## 3 Conclusions

In this study, the kinetics of the interactions between Cu^2+^ and WT‐αSyn, NAc‐αSyn and H50Q αSyn has been investigated. His50 of WT‐αSyn was determined to be irrelevant to high‐affinity Cu^2+^ binding from kinetic perspective. Whereas N‐terminal acetylation was found to significantly impact Cu^2+^ binding kinetics of αSyn. According to the Cu^2+^ binding affinity determined in this work, NAc‐αSyn (*K*_d_=23(3) μM) possesses around four orders of magnitude weaker affinity than WT‐αSyn (*K*_d_=3.0(7) nM). Such a result may connect to an important function of N‐terminal acetylation of αSyn, which is to prevent the binding of labile Cu^2+^ to abundant αSyn in the brain. In addition, WT‐αSyn‐Cu(II) complex was found to be reduction‐inactive towards ascorbate, but active in the presence of physiological concentration of GSH. In summary, the current study has provided new information about the interactions between Cu^2+^ and αSyn from a kinetic perspective. Controversy regarding the involvement of His50 for high‐affinity Cu^2+^ binding has been resolved and physiological significance of N‐terminal acetylation on the regulation of Cu^2+^ binding to αSyn has been proposed.

## Experimental Section

### α‐Synuclein Expression and Purification

WT‐αSyn was expressed and purified based on a protocol optimised from a previously published report.^[43]^ Plasmid pT7‐7 asyn WT (Addgene plasmid # 36046) was first transformed in BL21(DE3) *E. coli* via heat shock. The transformed BL21(DE3) *E. coli* cells were inoculated into 800 mL of LB containing 100 μg mL^−1^ ampicillin, and then incubated at 37 °C with 220 rpm shaking until the OD_600_ reached 0.7. After that, IPTG was added to a final concentration of 1 mM to induce WT‐αSyn expression. The cells were further incubated 3 h at 37 °C with 220 rpm shaking, and then harvested by centrifugation at 8000 g for 30 min at 4 °C. The cell pellet was resuspended in Tris‐HCl buffer (20 mM Tris‐HCl, 50 mM NaCl, 5 mM EDTA, pH 7.4). A protease inhibitor tablet (cOmplete, Roche) was dissolved in the suspension to protect protein from degradation before performing cell lysis. After 2 min cell lysis by ultrasound sonication, the suspension was boiled at 90 °C for 20 min and then centrifuged at 16000 g for 20 min. The supernatant was collected and filtered by 0.2 μm syringe filter to remove all cell debris. Subsequently, streptomycin sulfate was added to the supernatant to a final concentration of 10 mg mL^−1^, and the mixture was stirred for 15 min at 4 °C to precipitate nucleic acids. After centrifugation at 16000 g for 20 min, the supernatant was collected and ammonium sulfate was added to 50 % saturation. The mixture was stirred for 30 min at 4 °C and centrifuged again at 16000 g. Then the pellet was collected and resuspended in Tris‐HCl buffer (20 mM Tris‐HCl, 50 mM NaCl, pH 7.4) and dialysed overnight with 2 L of the same buffer. Protein concentration was determined from the absorbance at 280 nm with an extinction coefficient of 5960 M^−1^ cm^−1^ using a UV‐Vis spectrometer. The purified WT‐αSyn was characterised by ESI‐MS (Figure S8a). The final sample was aliquoted, flash frozen in liquid nitrogen and stored at −80 °C.

The analogue methods were used to produce NAc‐αSyn. pT7‐7 asyn WT plasmid was transformed together with pNatB (pACYCduet‐naa20‐naa25) plasmid (Addgene plasmid # 53613) into BL21(DE3) *E. coli*. The transformed BL21(DE3) *E. coli* cells were inoculated into 800 mL of LB containing 100 μg mL^−1^ ampicillin and 25 μg mL^−1^ chloramphenicol for NAc‐αSyn expression. The same expression and purification procedures were carried out as described for WT‐αSyn above. Purified NAc‐αSyn was characterised by ESI‐MS (Figure S8b).

A histidine to glutamine at position 50 of αSyn was introduced using a Phusion Site‐Directed Mutagenesis Kit (Thermo Fisher Scientific, Massachusetts, USA) following the manufacturer's protocol to produce H50Q αSyn. The primers and the sequencing result of mutated plasmid are shown in Figure S9a. The expression and purification of H50Q αSyn were carried out using the analogue methods as described above.

### α‐Synuclein Labelling

A glycine to cysteine mutation was introduced at position 7 of αSyn samples using a Phusion Site‐Directed Mutagenesis Kit (Thermo Fisher Scientific, Massachusetts, USA) for site‐specific labelling. The primers and the sequencing result of mutated plasmid are shown in Figure S9b. Dye‐labelling of αSyn was achieved via a selective thiol‐maleimide reaction. Following on protein production, αSyn samples were labelled with Alexa Fluor 488 C_5_ maleimide (Thermo Fisher Scientific, Massachusetts, USA) according to the instruction provided by the manufacturer. Briefly, 10 mM dye stock solution was pre‐prepared in dimethylsulfoxide and mixed with disulfide bond reduced G7C‐αSyn solution to a final molar ratio of 3 : 1 (dye : protein). The mixture was stirred in the dark for 3 h. Then the mixture was desalted using a PD‐10 desalting column containing Sephadex G‐25 resin (GE Healthcare Life Sciences, Illinois, USA), and concentrated using 10 K MWCO pierce protein concentrators (Thermo Fisher Scientific, Massachusetts, USA) to remove unreacted free dye. The final labelled αSyn concentration was determined from the absorbance at 495 nm with an extinction coefficient of 72000 M^−1^ cm^−1^ and the labelling efficiency was determined to be 95 %. The labelled αSyn samples were stored at −80 °C.

### Stopped Flow Kinetics

All kinetic measurements were performed on a KinetAsyst SF‐610X2 stopped flow spectrophotometer (HI‐TECH Scientific, UK). Samples were excited by a fibre‐coupled MCLS1‐473‐20 diode laser at 473 nm (Thorlabs, USA). Fluorescence emission was filtered using a 515 nm long pass filter (Comar, UK) before being detected by a photon multiplier tube. Data were recorded using a logarithmic time‐scale sampling scheme, and a minimum of 9 repeats were averaged. Data points below 2 ms were excluded in analysis to avoid the influence of the instrument dead time.

### EPR Spectroscopy Measurements

CW EPR spectra of αSyn‐Cu(II) complexes were detected with a Bruker EMX 300 EPR spectrometer equipped with a high sensitivity X‐band (ca. 9.4 GHz) resonator and a liquid helium cryostat. Field corrections were applied by measuring relevant EPR standards (Bruker Strong Pitch and DPPH). For accuracy, the tube size and tube position in the cavity was kept constant. Sample solution was transferred into an EPR tube (4 mm o.d.) via micropipettes then the tube was placed into 5 mm o.d. tube which was perched with argon gas and sealed by a silicone plug. Then the sample was frozen in liquid nitrogen and transferred into cryostat to cool down to 20 K. CW EPR spectra were recorded at a microwave power of ∼7 mW, modulation frequency of 100 kHz, and modulation amplitude of 10 G. Simulation of the EPR spectra was performed with the EasySpin/MATLAB toolbox, which employs the exact diagonalisation of the spin Hamiltonian matrix.^[44]^

### Kinetic Data Analysis

The averaged raw curves were analysed using OriginPro 2015 (OriginLab, USA). Reaction curves obtained from the measurements under 1 : 1 stoichiometric ratio of αSyn to Cu^2+^ were fitted to Equation (1) which was derived from second‐order reaction rate equation to obtain reaction rate *k*,

(1) $${\left[A\right]}_{t}=\frac{A}{Akt+1}+C$$

where *t* is the reaction time, [A]_*t*_ is the concentration of αSyn at time *t*, *A* is the amplitude of trace and *C* is the baseline value.

Reaction curves obtained from the measurements under pseudo first‐order conditions were fitted to Equation (2), a double exponential function,

(2) $${\left[A\right]}_{t}=A_{1}e^{-k_{1}t}+A_{2}e^{-k_{2}t}+C$$

Mean rate (*k*_mean_) values were calculated by Equation (3), and standard errors were calculated by Equation (4). In order to determine Cu^2+^ binding rate constant *k*_on_, *k*_mean_ values at different HEPES concentrations were empirically fitted with a parabola centred at zero.

(3) $$k_{mean}=\frac{A_{1}k_{1}+A_{2}k_{2}}{A_{1}+A_{2}}$$

(4) $$\sigma_{F}^{2}={\left(\frac{\partial F}{\partial A_{1}}\right)}^{2}\cdot \sigma_{A_{1}}^{2}+{\left(\frac{\partial F}{\partial k_{1}}\right)}^{2}\cdot \sigma_{k_{1}}^{2}+{\left(\frac{\partial F}{\partial A_{2}}\right)}^{2}\cdot \sigma_{A_{2}}^{2}+{\left(\frac{\partial F}{\partial k_{2}}\right)}^{2}\cdot \sigma_{k_{2}}^{2}$$

## Conflict of interest

The authors declare no conflict of interest.

## Supporting information

As a service to our authors and readers, this journal provides supporting information supplied by the authors. Such materials are peer reviewed and may be re‐organized for online delivery, but are not copy‐edited or typeset. Technical support issues arising from supporting information (other than missing files) should be addressed to the authors.

Supporting Information
