# Supplementary material for: Acetylation Rather than H50Q Mutation Impacts the Kinetics of Cu(II) Binding to α‐Synuclein
Source: Chemphyschem. 2021 Oct 14;22(23):2413–9. doi: 10.1002/cphc.202100651 (PMC9293329; doi:10.1002/cphc.202100651)
Supplement: Supplementary file 1 — Supporting Information [file CPHC-22-2413-s001.pdf]

ChemPhysChem

Supporting Information

Acetylation Rather than H50Q Mutation Impacts the Kinetics of Cu(II) Binding to α-Synuclein

Xiangyu Teng, Alena Sheveleva, Floriana Tuna, Keith R. Willison, and Liming Ying*

Supporting Information

Table of Contents

Figure S1. Comparison of the Cu^{2+} binding traces between WT-αSyn and H50Q WT-αSyn. 3

Figure S2. Comparison of the reaction kinetics of EDTA between the Cu^{2+} coordinated complexes of WT-αSyn and H50Q WT-αSyn. 4

Figure S3. Reaction traces of WT-αSyn-Cu(II) with EDTA at different pH. 5

Figure S4. The Peisach-Blumberg plot. 6

Figure S5. Kinetics of Cu^{2+} binding to αSyn. 7

Figure S6. Kinetics of Cu^{2+} extraction from αSyn-Cu(II) complexes by EDTA. 8

Figure S7. Reduction kinetics of WT-αSyn-Cu(II) complex. 9

Figure S8. ESI-MS characterisation of purified αSyn. 10

Figure S9. Mutation analysis by Sanger sequencing. 11

Figure S10. CD spectra of WT-αSyn-Cu(II) and Alexa 488 labelled WT-αSyn-Cu(II). 12

Table S1. $g_{\parallel}$ factors and hyperfine coupling constants ($A_{\parallel}$) for the EPR spectra of NAc-αSyn-Cu(II) and WT-αSyn-Cu(II). 6

Reference 12

Full sequencing results 13

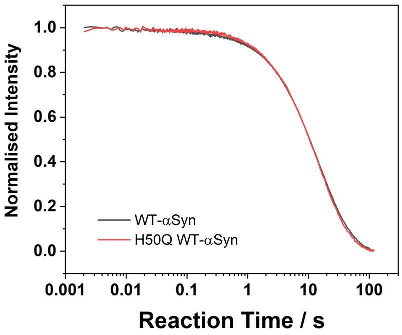

[αSyn, Cu^{2+}] = 100 nM

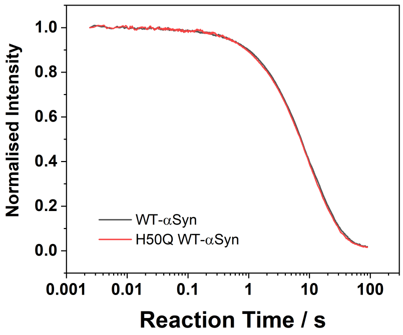

[αSyn, Cu^{2+}] = 150 nM

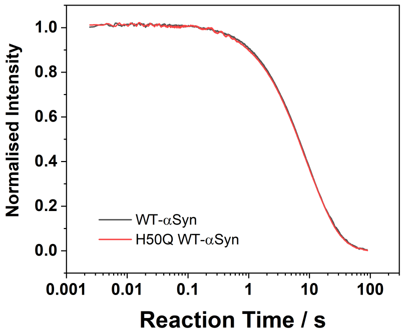

[αSyn, Cu^{2+}] = 200 nM

Figure S1. Comparison of the Cu^{2+} binding traces between WT-αSyn and H50Q WT-αSyn. The measurements were performed under different concentrations. All measurements were performed in 50 mM HEPES buffer with 100 mM NaCl at 298 K (pH 7.5).

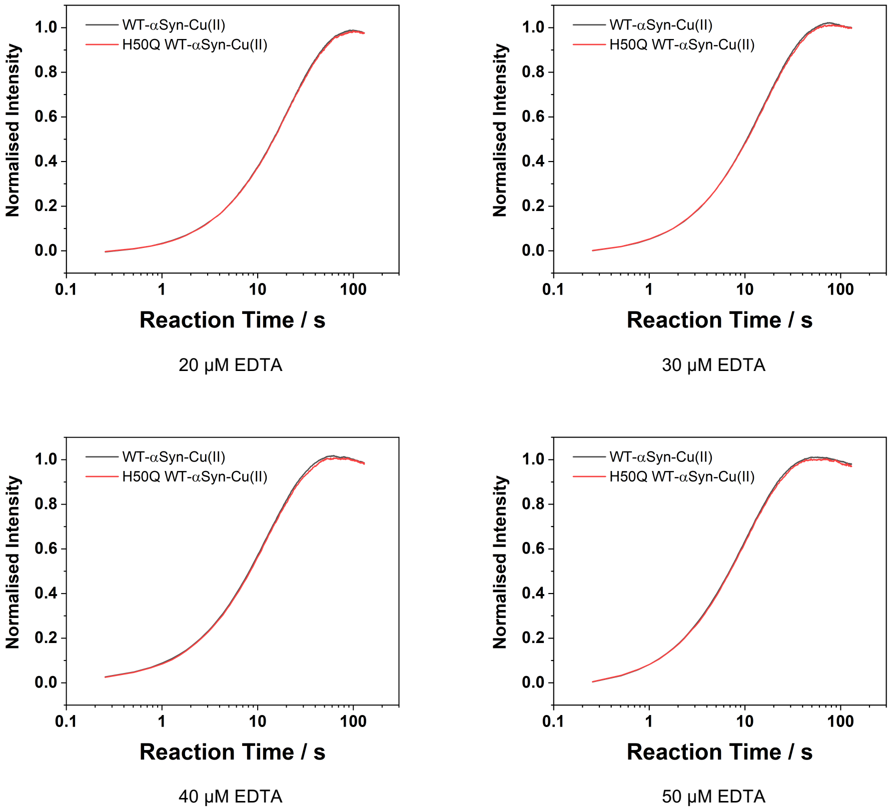

Figure S2. Comparison of the reaction kinetics of EDTA between the Cu^{2+} coordinated complexes of WT-αSyn and H50Q WT-αSyn. The measurements were performed under different EDTA concentrations. All measurements were performed in 50 mM HEPES buffer with 100 mM NaCl at 298 K (pH 7.5).

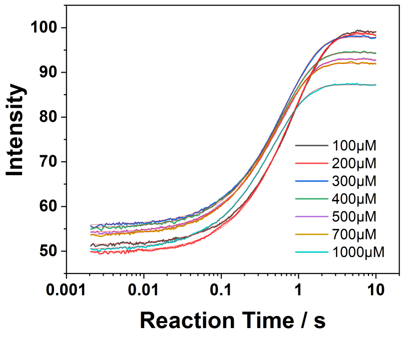

pH 5.5

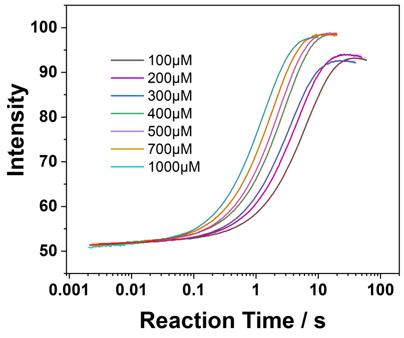

pH 6.5

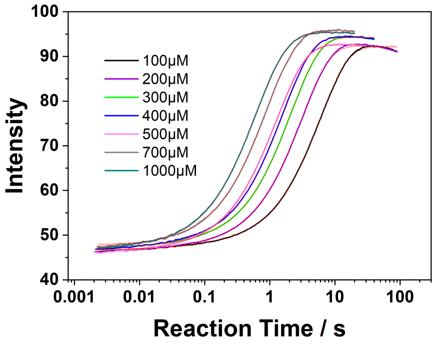

pH 7.5

Figure S3. Reaction traces of WT-αSyn-Cu(II) with EDTA at different pH. Raw traces and fitted curves (shown as red solid curves) of WT-αSyn-Cu(II) dissociation under various concentrations of EDTA at different pH. All of the traces can be fitted perfectly by single exponential fit, suggesting that only one coordination species is present. The experiments were performed in 50 mM HEPES buffer with 100 mM NaCl at 298 K.

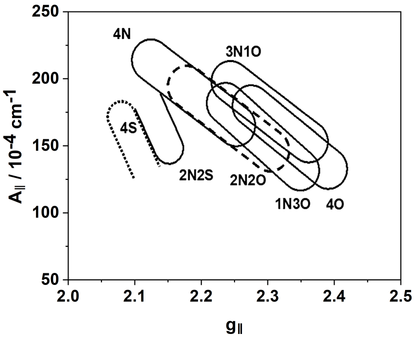

Figure S4. The Peisach-Blumberg plot. The plot showing the correlation between coordinating atoms of Cu^{2+} and EPR parameters (adapted from Ref 1). $A_{\parallel}$ in different units were transformed by the equation $A(\mathrm{cm}^{-1}) = 0.46686451 \times 10^{-4}\, gA(\mathrm{G})$[1-3].

Table S1. $g_{\parallel}$ factors and hyperfine coupling constants ($A_{\parallel}$) for the EPR spectra of NAc-αSyn-Cu(II) and WT-αSyn-Cu(II). Data obtained from simulation in Figure 3b.

	$g_{\parallel}$ factor	$A_{\parallel}$ / G
NAc-αSyn-Cu(II)	2.29	195
WT-αSyn-Cu(II)	2.245	208

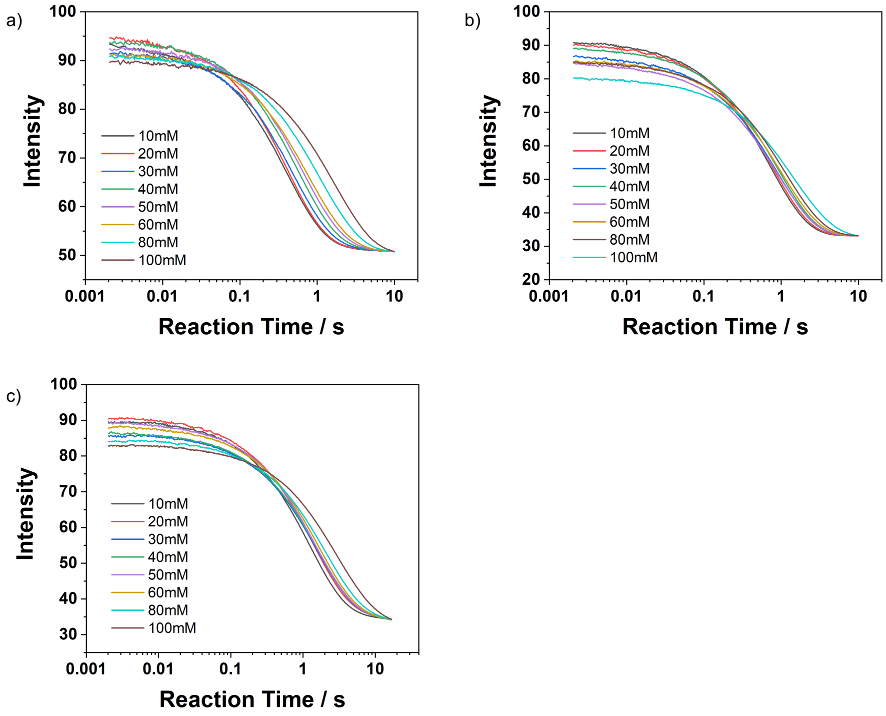

Figure S5. Kinetics of Cu^{2+} binding to αSyn. Raw traces of Cu^{2+} binding to a) WT-αSyn, b) NAc-αSyn and c) H50Q NAc-αSyn under various concentrations of HEPES. The experiments were performed in HEPES buffer with 100 mM NaCl at 298 K (pH 7.5).

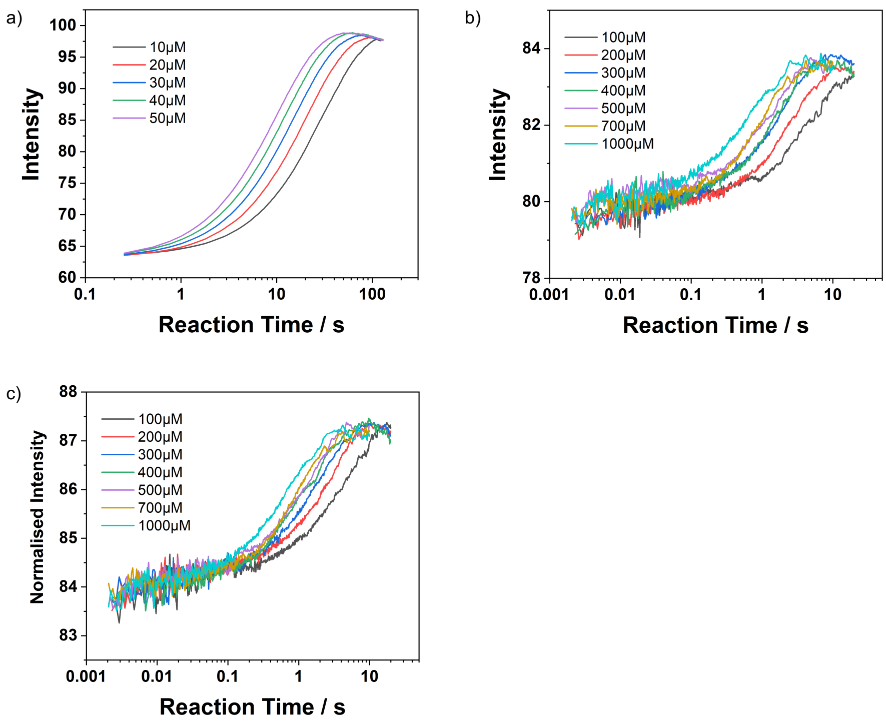

Figure S6. Kinetics of Cu^{2+} extraction from αSyn-Cu(II) complexes by EDTA. Raw traces of Cu^{2+} extraction from a) WT-αSyn-Cu(II), b) NAc-αSyn-Cu(II) and c) H50Q NAc-αSyn-Cu(II) under various concentrations of EDTA. The experiments were performed in 50 mM HEPES buffer with 100 mM NaCl at 298 K (pH 7.5).

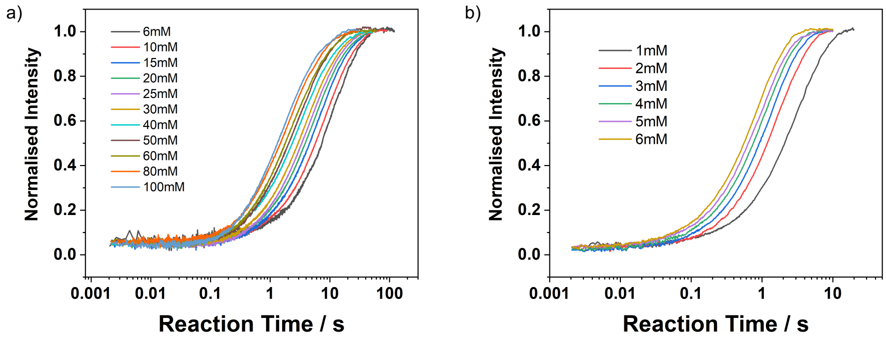

Figure S7. Reduction kinetics of WT-αSyn-Cu(II) complex. Reduction traces of WT-αSyn-Cu(II) by various concentrations of a) ascorbate and b) GSH. The experiments were performed in 50 mM HEPES buffer with 100 mM NaCl at 298 K (pH 7.5).

a)

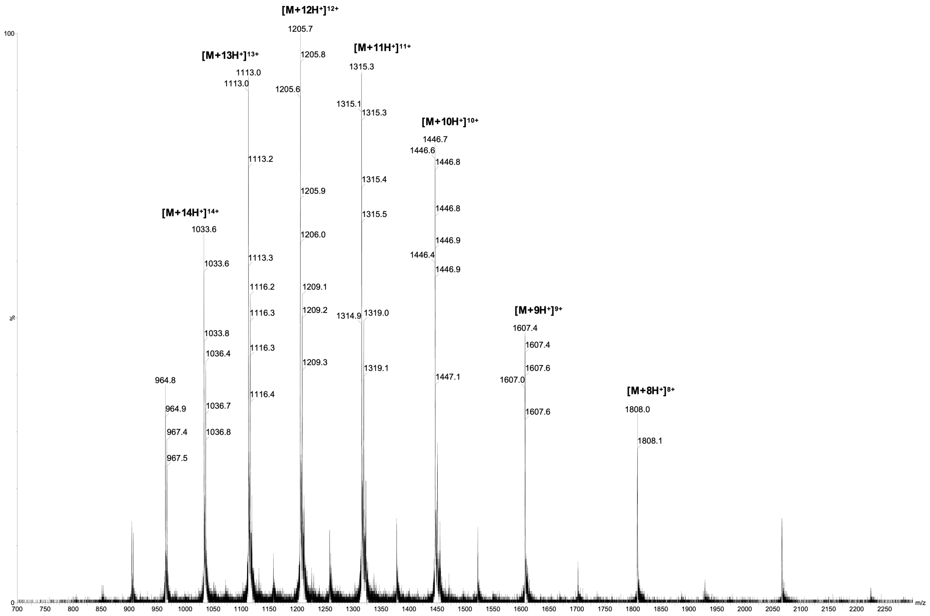

b)

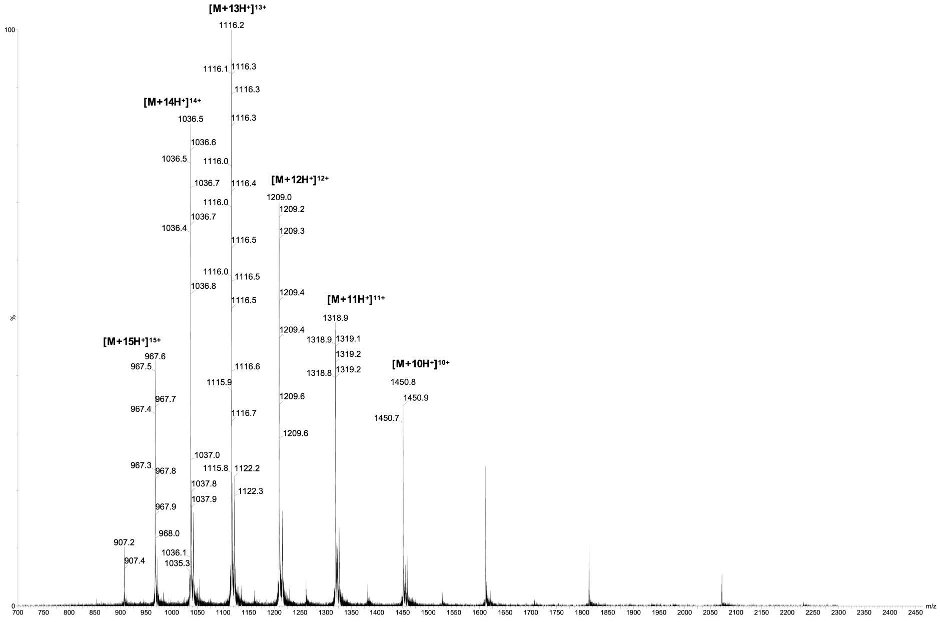

Figure S8. ESI-MS characterisation of purified a) WT-αSyn and b) NAc-αSyn. Molecular masses were calculated as: $m/z = [M + nH^{+}] / n^{+}$, $M = (m/z \times n) - n$. The measured molecular masses of WT-αSyn and NAc-αSyn are 14456 Da and 14499 Da, respectively. A 43 Da difference of molecular mass indicates the formation of an acetyl group on WT-αSyn molecule.

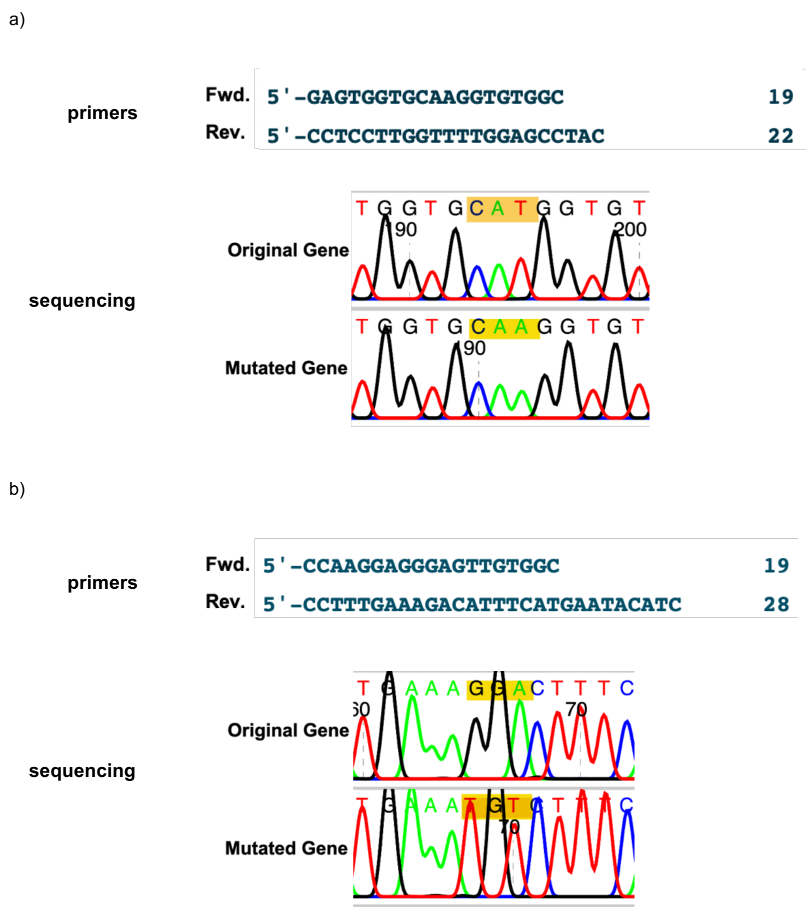

Figure S9. Mutation analysis by Sanger sequencing. a) Designed primers and sequencing result of H50Q mutation of WT-αSyn. The result showing a successful codon substitution of histidine 50 (CAT) to glutamine (CAA). b) Designed primers and sequencing result of G7C mutation of WT-αSyn for labelling. The result showing a successful codon substitution of glycine 7 (GGA) to cysteine (TGT). The full sequencing results are attached on the following pages.

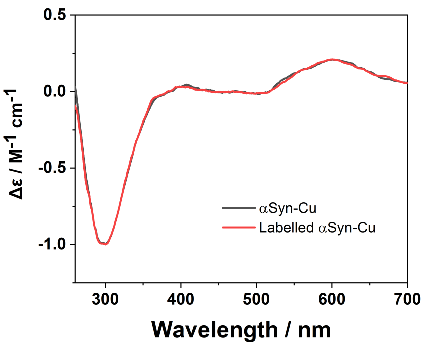

Figure S10. CD spectra of WT-αSyn-Cu(II) and Alexa 488 labelled WT-αSyn-Cu(II). The identical curves indicate that the attached Alexa 488 dye does not impact Cu^{2+} coordination geometry of αSyn. The experiments were performed in 50 mM HEPES buffer with 100 mM NaCl at 298 K (pH 7.5).

Reference

[1] J. Peisach, W. Blumberg, *Arch. Biochem. Biophys.* **1974**, *165*, 691-708.

[2] J. F. Boas, S. C. Drew, C. C. Curtain, *Eur. Biophys. J.* **2008**, *37*, 281-294.

[3] M. Brustolon, E. Giamello in *Electron paramagnetic resonance: a practitioner’s toolkit* (Eds.: M. Brustolon, E. Giamello), John Wiley & Sons, Inc., New Jersey, **2009**, pp. 520-522.

GENEWIZ
Solid science. Superior service.

H50Q mutation sequencing (control)

Sequence: G7C-A-SYN_T7P_1
Order: COL17-05US

Machine: ZC-1402-007
Signal: A (492), C (419), G (752), T (380)

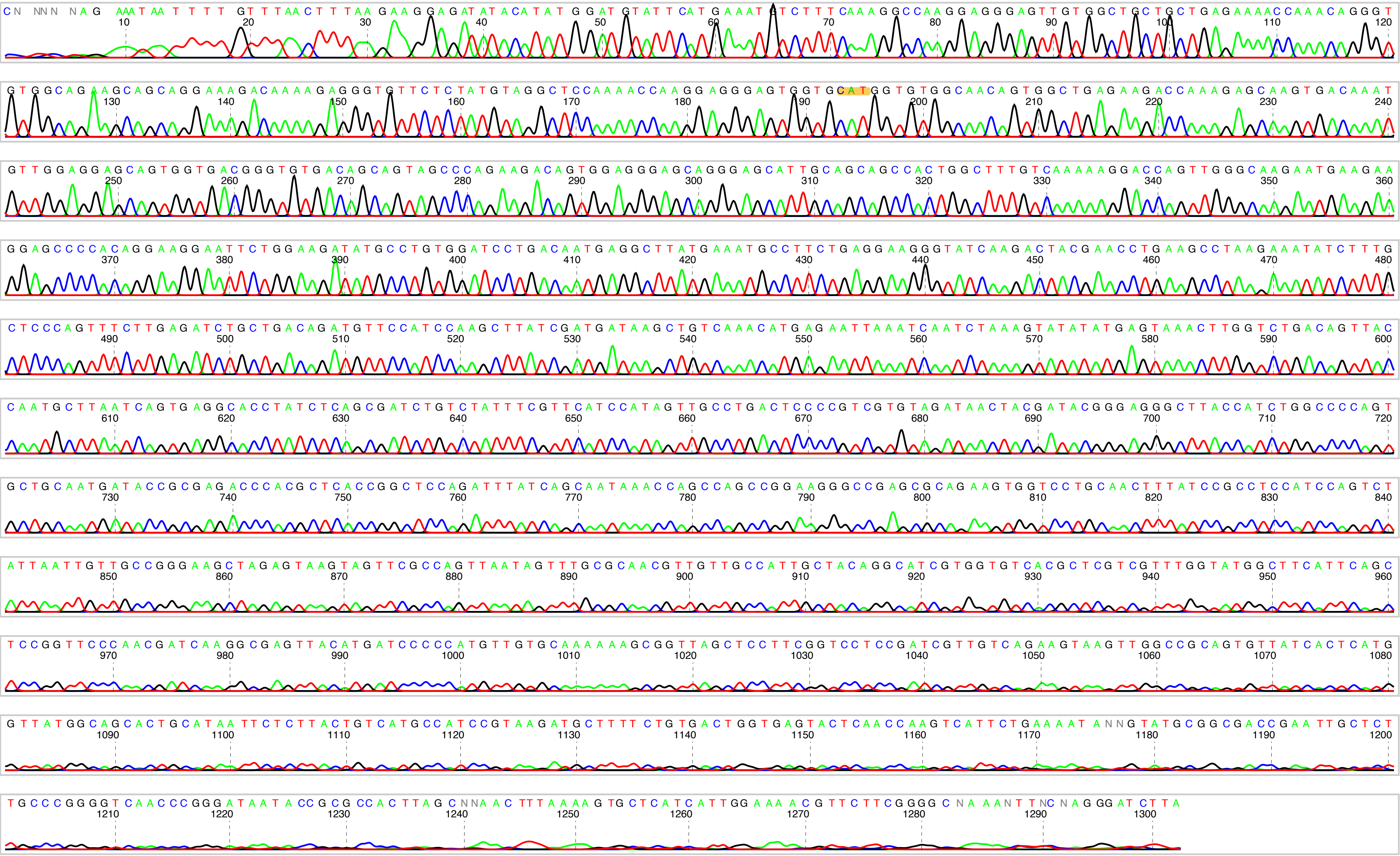

Cogenics Online, 20 June, 2017 19:20

H50Q mutation sequencing (mutated)

Sequence: H50Q-A-SYN_T7P_2
Order: COL17-05US

Machine: ZC-1402-007
Signal: A (748), C (620), G (989), T (576)

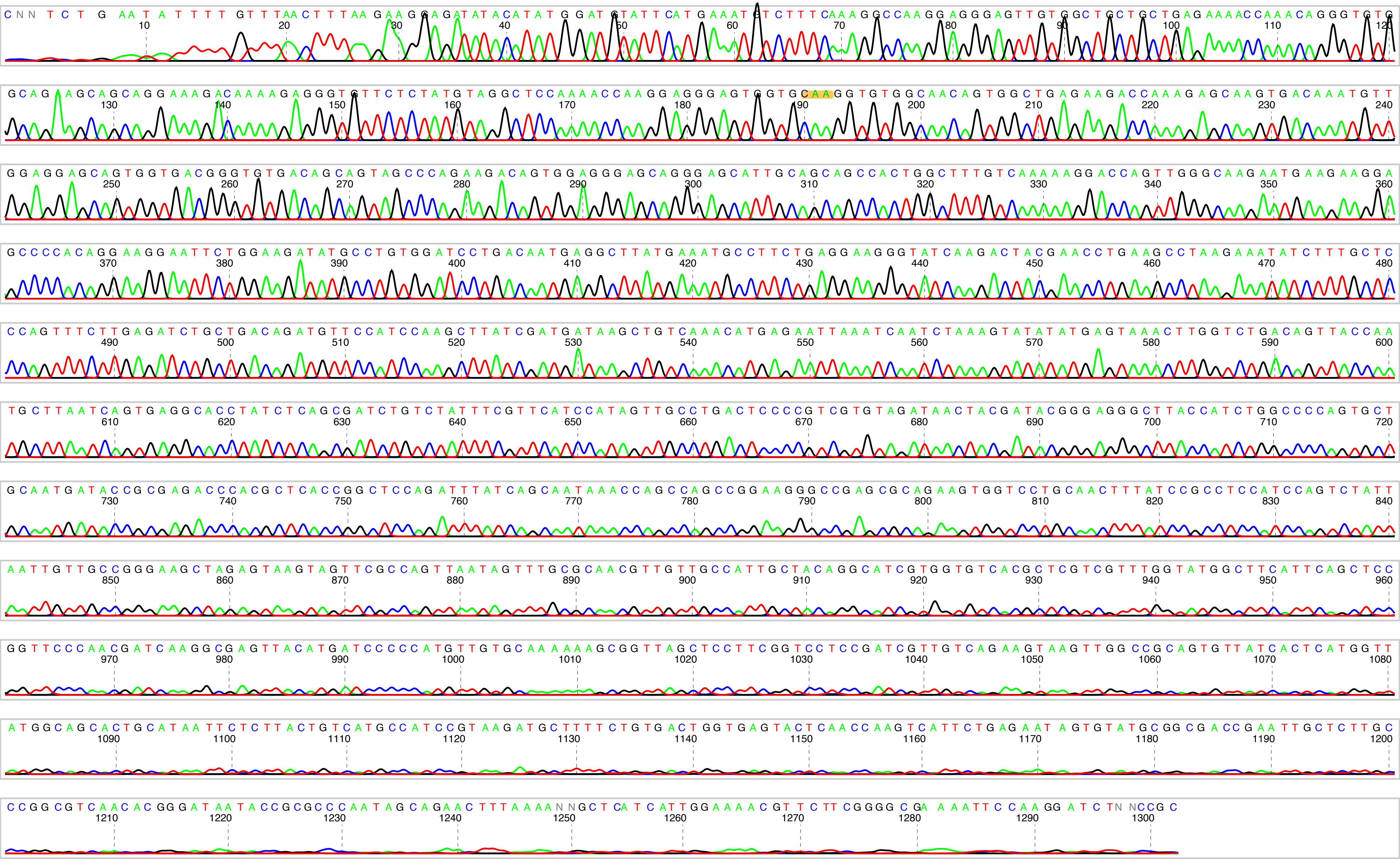

Cogenics Online, 20 June, 2017 19:20

GENEWIZ®
Solid science. Superior service.

G7C mutation sequencing (control)

Sequence: A-SYN-WT_T7P_1
Order: COL17-04RF

Machine: CHIGNIN-1403-019
Signal: A (3529), C (3317), G (2183), T (2333)

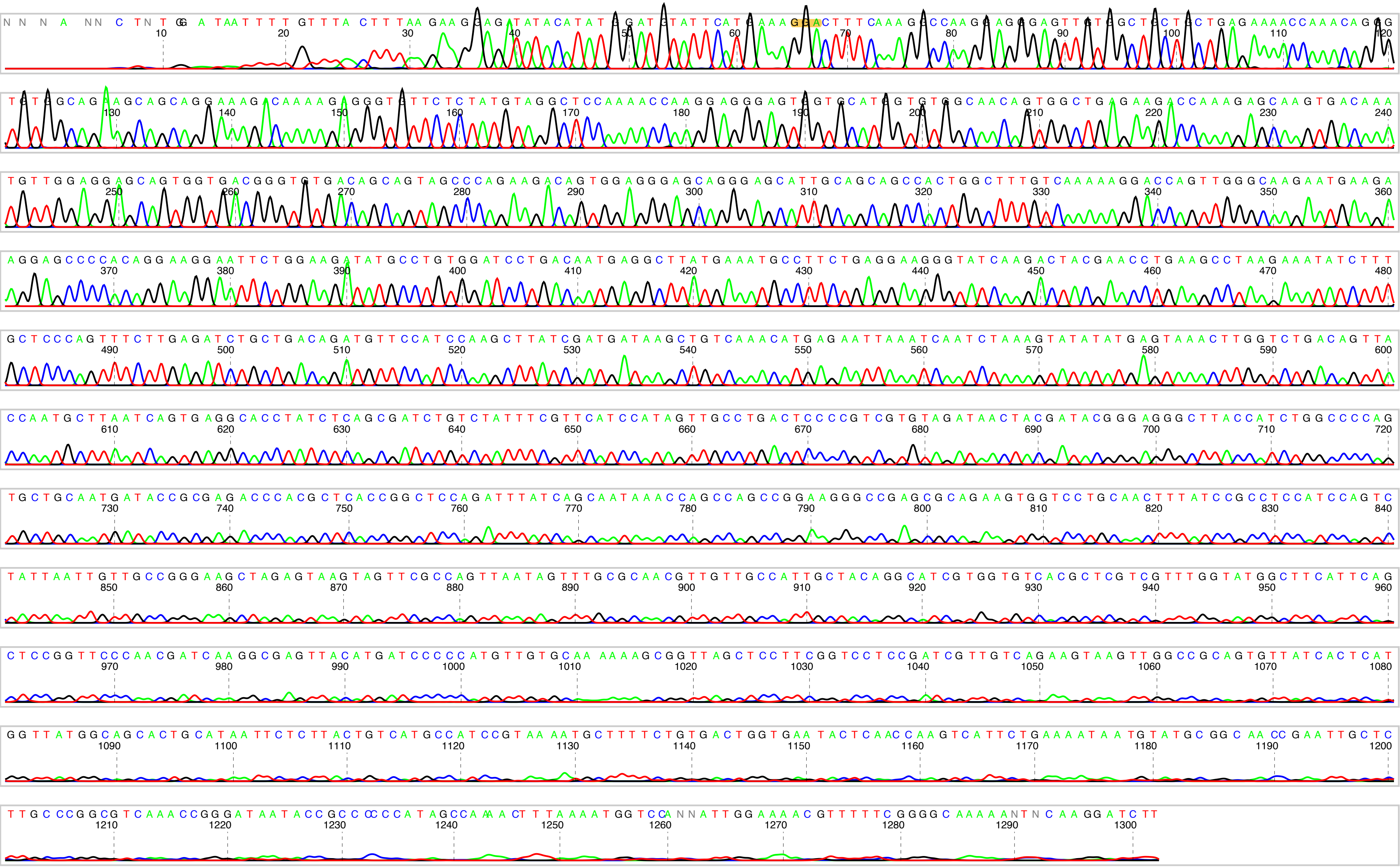

Cogenics Online, 18 May, 2017 13:34

G7C mutation sequencing (mutated)

GENEWIZ®
Solid science. Superior service.

Sequence: A-SYN-G7C_T7P_2
Order: COL17-04RF

Machine: CHIGNIN-1403-019
Signal: A (3112), C (2559), G (2452), T (2092)

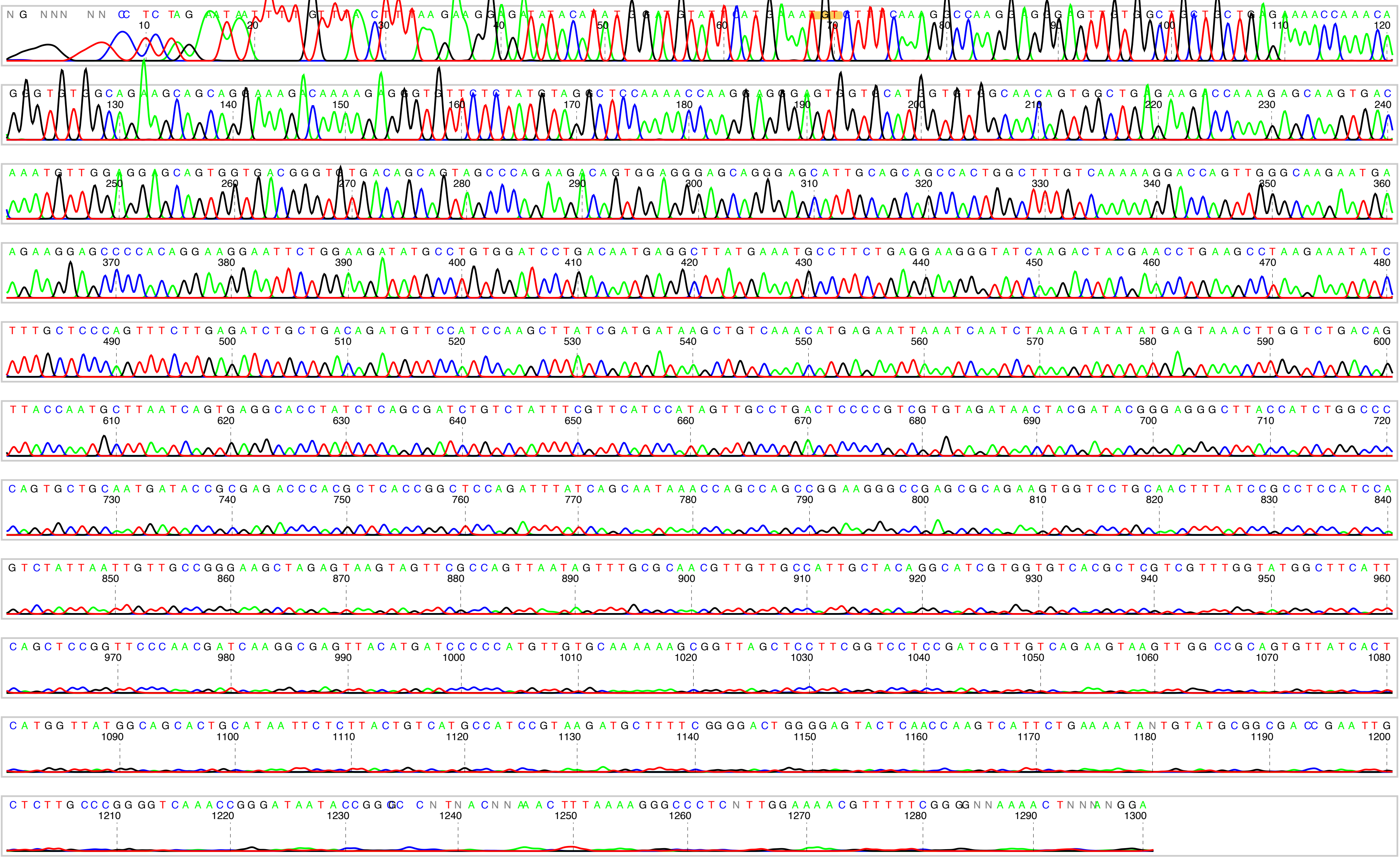

Cogenics Online, 18 May, 2017 13:34
